# Proteomic and Transcriptomic Analysis Identify Spliceosome as a Significant Component of the Molecular Machinery in the Pituitary Tumors Derived from *POU1F1*- and *NR5A1*-Cell Lineages

**DOI:** 10.3390/genes11121422

**Published:** 2020-11-27

**Authors:** Keiko Taniguchi-Ponciano, Eduardo Peña-Martínez, Gloria Silva-Román, Sandra Vela-Patiño, Ana Laura Guzman-Ortiz, Hector Quezada, Erick Gomez-Apo, Laura Chavez-Macias, Sophia Mercado-Medrez, Guadalupe Vargas-Ortega, Ana Laura Espinosa-de-los-Monteros, Baldomero Gonzales-Virla, Aldo Ferreira-Hermosillo, Etual Espinosa-Cardenas, Claudia Ramirez-Renteria, Ernesto Sosa, Blas Lopez-Felix, Gerardo Guinto, Daniel Marrero-Rodríguez, Moises Mercado

**Affiliations:** 1 Unidad de Investigación Medica en Enfermedades Endocrinas, Hospital de Especialidades, Centro Medico Nacional Siglo XXI, Instituto Mexicano del Seguro Social, Av. Cuauhtémoc 330, Col. Doctores, Mexico D.F. 06720, Mexico; keiko.taniguchi@hotmail.com (K.T.-P.); juaneduardomartinez300@gmail.com (E.P.-M.); gloriasil44@gmail.com (G.S.-R.); sanvela9231@gmail.com (S.V.-P.); sopmer99@gmail.com (S.M.-M.); aldo.nagisa@gmail.com (A.F.-H.); clau.r2000@gmail.com (C.R.-R.); 2 Laboratorio de Investigacion en Inmunologia y Proteomica, Hospital Infantil de Mexico “Federico Gomez”, Mexico City 06720, Mexico; lauris_26san@hotmail.com (A.L.G.-O.); hquezadap@yahoo.com.mx (H.Q.); 3 Área de Neuropatología, Servicio de Anatomía Patológica, Hospital General de México “Dr. Eduardo Liceaga”, Ciudad de México 06720, Mexico; erickapo@hotmail.com (E.G.-A.); laurachm@prodigy.net.mx (L.C.-M.); 4 Facultad de Medicina, Universidad Nacional Autonoma de México, Mexico City 04510, Mexico; 5 Servicio de Endocrinologia, Hospital de Especialidades, Centro Medico Nacional Siglo XXI, Instituto Mexicano del Seguro Social, Ciudad de México 06720, Mexico; gvargas_ortega@hotmail.com (G.V.-O.); analems@hotmail.com (A.L.E.-d.-l.-M.); baldogonzal@hotmail.com (B.G.-V.); espinosaetual@gmail.com (E.E.-C.); esosae@yahoo.com (E.S.); 6 Servicio de Neurocirugia, Hospital de Especialidades, Centro Medico Nacional Siglo XXI, Instituto Mexicano del Seguro Social, Ciudad de México 06720, Mexico; belopfel@gmail.com (B.L.-F.); gguinto@prodigy.net.mx (G.G.); 7 Catedra CONACyT-Laboratorio de Endocrinologia Experimental, Unidad de Investigación Medica en Enfermedades Endocrinas, Hospital de Especialidades, Centro Medico Nacional Siglo XXI, Instituto Mexicano del Seguro Social, Av. Cuauhtémoc 330, Col. Doctores, Mexico D.F. 06720, Mexico

**Keywords:** pituitary adenomas, alternative splicing, molecular markers, mRNA isoforms

## Abstract

Background: Pituitary adenomas (PA) are the second most common tumor in the central nervous system and have low counts of mutated genes. Splicing occurs in 95% of the coding RNA. There is scarce information about the spliceosome and mRNA-isoforms in PA, and therefore we carried out proteomic and transcriptomic analysis to identify spliceosome components and mRNA isoforms in PA. Methods: Proteomic profile analysis was carried out by nano-HPLC and mass spectrometry with a quadrupole time-of-flight mass spectrometer. The mRNA isoforms and transcriptomic profiles were carried out by microarray technology. With proteins and mRNA information we carried out Gene Ontology and exon level analysis to identify splicing-related events. Results: Approximately 2000 proteins were identified in pituitary tumors. Spliceosome proteins such as *SRSF1*, *U2AF1* and *RBM42* among others were found in PA. These results were validated at mRNA level, which showed up-regulation of spliceosome genes in PA. Spliceosome-related genes segregate and categorize PA tumor subtypes. The PA showed alterations in *CDK18* and *THY1* mRNA isoforms which could be tumor specific. Conclusions: Spliceosome components are significant constituents of the PA molecular machinery and could be used as molecular markers and therapeutic targets. Splicing-related genes and mRNA-isoforms profiles characterize tumor subtypes.

## 1. Background

Pituitary adenomas (PA) account for 10–25% of the total intracranial tumors, and despite their very low malignancy rate they cause significant morbidity [1]. Pituitary tumors are classified immunohistochemically according to their hormone profile and transcription factor expression. There are three main transcription factors determining terminal differentiation of the anterior pituitary cell lineages: *POU1F1*, controls somatotrope, thyrotrope and lactotrope differentiation and is expressed in *GH*-, *TSH*- and *PRL*-secreting tumors; *NR5A1*, controls gonadotrope differentiation and is expressed in most clinically non-functioning pituitary adenomas (NFPA) which produce *LH* and/or *FSH*; and *TBX19*, which dictates corticotrope differentiation and is expressed in ACTH-producing tumors causing Cushing disease and in silent *ACTH* adenomas [2]. The molecular basis of the pituitary tumorigenesis is largely unknown since gene mutations are seldom observed in this kind of tumor [1], thus, other molecular mechanisms are likely to be involved in pituitary tumorigenesis [3].

Splicing of mRNA precursors is required for the maturation of almost all human mRNA’s and is a key step in the regulation of the expression of many genes [4]. Alternative splicing is a ubiquitous regulatory mechanism of gene expression that allows generation of more than one unique mRNA species from a single gene resulting in the synthesis of different protein isoforms. Approximately 90–95% of the genome undergoes some degree of alternative splicing which contributes to cell differentiation, lineage determination tissue identity and organ maintenance and development [5]. Several tumor transcriptomes have revealed that they exhibit aberrant splicing, including changes in the usage of annotated transcript isoforms and the use of unannotated splicing events [6]. Splicing in tumors can affect crucial genes related to processes such as angiogenesis, apoptosis, as well as cellular energetics and proliferation [7].

Information regarding splicing-related events in PA and components information is scarce, with genes such as *DIO1* [1] and *CSH2* [8] showing mRNA isoforms. In the present work we carried out proteomic and transcriptomic analyses to identify altered genes related to splicing events in pituitary adenomas derived from *POU1F1* and *NR5A1* cell lineages.

## 2. Materials and Methods

### 2.1. Pituitary Tissue Collection

For the proteome analysis, 20 pituitary tissue samples were collected, including 6 non-tumoral pituitaries used as controls; 8 NFPA, 4 *GH*-secreting adenomas; one *TSH*-secreting adenoma and one prolactinoma. For the transcriptome analysis 42 pituitary tissue samples were collected, including 6 non-tumoral pituitaries, 20 NFPA (14 gonadotropinomas, 3 null cell adenomas and 3 silent *ACTH*-producing adenomas), 10 somatotropinomas, 4 thyrotropinomas and two prolactinomas. Tumors were collected at the time of surgery from patients diagnosed, treated and followed at the Neurosurgery and Endocrinology departments of the Hospital de Especialidades-CMNSXXI, whereas the control, non-tumoral pituitary glands were obtained from autopsies performed no more than 10 h after patient death at the Pathology department of the Hospital General de México, both in México City, between May 2016 and May 2018. All tissues were collected prior to the administration of any medical therapy and/or radiotherapy, when possible. Detailed clinical, biochemical, pathological and imaging information was obtained from the hospital’s electronic records. All participating patients were recruited with signed informed consent and ethical approval from the Comisión Nacional de Ética e Investigación Científica del Instituto Mexicano del Seguro Social in accordance with the Helsinki declaration.

### 2.2. Protein Purification

The Plasma Membrane Protein Extraction Kit (Abcam, Cambridge, UK) was used according to manufacturer’s specifications. Briefly, tissues were washed in ice-cold phosphate buffered saline (PBS) 1X as many times were needed to eliminate most of the blood present. The tissue was homogenized in 2 mL of ice-cold Homogeneize Buffer with Halt Protease and Phosphatase Inhibitor Cocktail 1X (Thermo, Waltham, MA, USA) in a BeadBug Microtube homogenizer (Benchmark, Sayreville, NJ, USA). The homogenate was centrifuged at 700× *g* for 10 min at 4 °C to collect tissue and cells that were not lysed. The supernatant was transferred to a new tube and was centrifuged at 10,000× *g* during 30 min at 4 °C. Pellet formed correspond to all membrane proteins and supernatant correspond to cytosolic proteins. Cytosolic proteins were precipitated with four volumes of 95% acetone and centrifuged.

### 2.3. Sample Preparation for Proteomic Analyses 

The proteins were dissolved in 20 μL of 0.2% Protease Max Surfactant (Promega, Madison, WI, USA) in 50 mM NH4HCO3 and 15 μL of UREA 8 M (Sigma-Aldrich, St. Louis, MO, USA). The equivalent to 200 μg of protein was reduced with 10 mM dithiothreitol (Sigma-Aldrich) at 37 °C for 60 min and alkylated with 20 mM iodoacetamide (Sigma-Aldrich), for 30 min at room temperature under the dark, then Tris-HCl pH 8.6 (Promega) was added to reach 10 mM. Digestion was made with trypsin (Promega) 1:35 at 37 °C overnight and then peptides were fractionated with HyperSep SCX cartridges (Thermo Scientific, Waltham, MA, USA) following the manufacturer’s instructions. Five fractions were obtained from each sample which were desalted with Sep-Pak tC18 cartridges (Waters, Milford, MA, USA), dried in a SpeedVac concentrator (Eppendorf, Hamburg, DE), and kept at −80 °C. The samples were reconstituted in 30 µL of 0.1% formic acid and 5% acetonitrile, centrifuged at 20,000× *g* at 4 °C for 5 min and injected on a C18 Nano HPLC column for separation of peptides [9].

### 2.4. Nano-HPLC-MS/MS Analysis

The peptide solutions (5 µL) were loaded into a Dionex UltiMate 3000 HPLC system (Thermo Scientific, Waltham, MA, USA) using a pre-column/peptide trap Acclaim PepMap 100 C18 (300 µm × 1.5 cm) (Dionex, Sunnyvale, CA, USA), and a separation column Acclaim PepMap RSLC C18 (75 µm × 15 cm) (Dionex, Sunnyvale, CA, USA). Chromatographic runs were performed at a constant flow of 300 nL/min of a mixture of 0.1% (*v/v*) formic acid in water (Buffer A, from a Milli-Q system), and 0.1% (*v/v*) formic acid in acetonitrile (Buffer B, HPLC grade from Sigma-Aldrich) in a linear gradient of 85 min from 2–40% B. At min 90, the gradient was increased to 90% B and was held there for 11 min after which the percentage of B was returned to 2% for column re-equilibration. Electrospray ionization of the eluted peptides was performed with a CaptiveSpray source (Bruker, Billerica, MA, USA) assisted by a flow of nitrogen boiled on acetonitrile (0.2 bar) and the mass spectra were acquired with a quadrupole time-of-flight mass spectrometer (Impact II, Bruker). Positive ions were analyzed over an *m/z* range of 100–2200. Before every six injections, calibration was performed with the electrospray ionization-time of flight (ESI-TOF) Tuning mix (Agilent, Santa Clara, CA, USA). MS/MS fragmentation was performed for those ions with a signal higher than 5000 counts applying a cycle time of 3 s and excluding +1 charged ions. Active exclusion was active after one spectrum for 2 min, unless the intensity of the precursor was more than three times higher than in the previous scan. Collision energy depended on the precursor ion charge and mass (e.g., at 700 *m/z*, 33 eV and 27 eV for 2+ and 3+ ions, respectively; whereas at 1100 *m/z*, 65 eV and 55 eV were used for 2+ and 3+ ions) [9].

### 2.5. Database Searching and Analysis of Proteomic Data

Protein identifications were made processing the raw files with the DataAnalysis-otof-default script from the Bruker Compass DataAnalysis software (version 4.4 SR1, Bruker, Billerica, MA, USA), the Protein Scape software (version 3.1.3 461, Bruker) using Mascot 2.4.1 (Matrix Science, City of Industry, CA, USA): trypsin as the digestion enzyme, two missed cleavages were allowed, carbamidomethyl Cys as a fixed modification and oxidation on Met as variable modification. Monoisotopic peptide masses were searched with 7.0 ppm peptide mass tolerance and 0.05 Da fragment mass tolerance. False discovery rate (FDR) was set to 1% with the peptide decoy and percolator options active. The SwissProt database for *Homo sapiens* was used. Proteins with Mascot scores >13 were considered as successful identifications [9].

### 2.6. RNA Purification

Total RNA was extracted from PA patients and pituitary control from autopsies using the miRNAeasy Mini Kit (Qiagen Inc, Hilden, DE, USA) according to manufacturer’s instructions. The pituitary tissue samples were disrupted and homogenized in 700 μL of Qiazol Lysis Reagent. They were then incubated at room temperature for 5 min. Next, 200 μL of chloroform was added, and samples were incubated at room temperature for 3 min. The mixture was centrifuged at 12,500 rpm for 15 min at 4 °C. The aqueous phase was transferred to a new tube and mixed with an equal volume of 70% ethanol. Samples were then transferred to an RNAeasy Column in a 2 mL tube, and centrifuged at 10,000 rpm for 15 s. After centrifugation, 700 μL of RW1 buffer was added and mixture was centrifuged at 10,000 rpm for 15 s. Flow-through was discarded and 500 μL of RPE buffer was added to the membrane and then centrifuged at 10,000 rpm for 15 s (2×). The column was transferred to a new collection tube then 30 μL of RNAse free water was added and centrifuged for 1 min at 10,000 rpm. RNA was quantified using a Nanodrop-ND-1000 (Thermo Scientific, Waltham, MA, USA) and RNA integrity was evaluated by Bioanalyzer 2100.

### 2.7. Microarray GeneChip Clariom D Assay

The microarray used for these studies was Affymetrix Clariom D which allows us to analyze the whole coding transcriptome at the gene and exon level as well as non-coding RNA such as lincRNA, miRNA and circRNA. Sample amplification and preparation for microarray hybridization was performed according to Affymetrix specifications according to the GeneChip WT Pico Reagent Kit protocol. Briefly, 100 ng of total RNA was reverse transcribed into cDNA, amplified by in vitro transcription and reversely transcribed to cDNA again. Fragments between 40 and 70 bp were generated enzymatically, labelled and hybridized onto the microarray chips in an Affymetrix hybridization oven at 60 rpms and 45 °C for 17 h. Chips were washed according to the stablished protocols (Affymetrix, Santa Clara, CA, USA) with a GeneChip fluidics station 450, and finally scanned with an Affymetrix 7G GeneChip scanner. The raw data (CEL files) were uploaded into the Gene Expression Omnibus (GEO), which is hosted by the National Center for Biotechnology Information (NCBI) under the accession number GSE147786.

### 2.8. Bioinformatic Analysis of the Pituitary Adenomas Transcriptome

A total of 6 control and 36 PA samples were analyzed, and two technical replicates. Data sets were analyzed by means of CEL files with the Expression Console, Partek Genomics Suite 6.6 v software (Partek Incorporated, Saint Louis, MO, USA) and Transcriptome Analysis Console (Affymetrix, Santa Clara, CA, USA). Pearson and Spearman correlations were performed and probe sets were summarized by means of Median Polish and normalized by quantiles with no probe sets excluded from analysis. Background noise correction was achieved by means of Robust Multi-chip Average (RMA) and data were log2- transformed. Data grouping and categorization was achieved by principal component analysis (PCA). Differentially expressed genes were determined by means of ANOVA. Genes were considered altered with +2- or −2-fold change, *p* ≤ 0.05 and FDR ≤ 0.05 parameters. The hierarchical clustering was carried out unsupervised, based in dissimilarity of samples (Euclidian method) by means of average linkage.

### 2.9. Immunohistochemical Analysis of Hormones and Transcription Factors

Paraffin-embedded, formalin-fixed tissue blocks were stained with hematoxylin-eosin and reviewed by a pathologist. Tumors were represented with 2-fold redundancy, which has been shown to provide a sufficiently representative sample. Sections (3 μm) were cut and placed onto coated slides. Slides were deparaffinized with xylene followed by ethanol and rehydrated. Immunostaining was performed using a HiDef detection HRP polymer system (Cell Marque, Rocklin, CA, USA). 

Briefly, after dewaxing the sections, endogenous peroxidase activity was inhibited. Next, the sections were processed in a 600 W microwave oven at maximum power, three times for 5 min each in Tris-EDTA buffer (pH 9.0). Incubation with antibodies against hormones (*ACTH* (M3501), *LH* (M3502), *FSH* (M3504); *PRL* (A0569), *GH* (A0570) Dako, Santa Clara, CA, USA, *TSH* (CM412B) BioCare Medical, Pacheco, CA, USA) and transcription factors (*NR5A1* (SC393592) Santa Cruz Biotechnology, Dallas, TX, USA, *TBX19* (AB243028), Abcam, CA, UK, *POU1F1* (NBP1-92273), Novus Biologicals, Centennial, CO, USA) was performed overnight at 4 °C in a humidity chamber at 1:100 dilutions, in 1% Bovine serum albumin (BSA). Sections were developed with a peroxidase substrate solution, counterstained with hematoxylin, dehydrated, and mounted. Control pituitary was used as positive biological controls, and negative controls consisted of the replacement of the primary antibody with 1% BSA. 

Two Independent pathologists reviewed the hormones and transcription factors expression by immunohistochemistry by light microscopy at 20× magnification. 

## 3. Results

### 3.1. Clinical Features from the Pituitary Adenomas and Control Tissue

A total of 50 pituitary tumor tissues were analyzed, 14 for proteomic analysis and 36 for transcriptome analysis. Most of the POU1F1- and NR5A1-derived tumors were macroadenomas (larger than 10 mm in diameter). Females predominated among patients harboring POU1F1-derived hormone secreting tumors (POU1F1), whereas there were no differences in gender in the NR5A1-derived clinically non-functioning PA. Cavernous sinus invasion was documented in 68.1% and 92.8% of POU1F1- and NR5A1-derived tumors, respectively. While 81.8% of the POU1F1-derived functioning adenomas were found to be recurrent upon follow up, that was the case for only 50% of the NR5A1-derived NFPA (Table 1). Supplementary Figure S1 shows a representative example of hormone and transcription factor immunohistochemistry.

From the 12 control gland obtained by autopsies, 6 for proteomic and 6 for transcriptomic analysis, 8 were from female and 4 from males. Age from the donors ranged between 21 and 78 years old. Death causes were among left ventricle hypertrophy, essential thrombocythemia and morbid obesity among the most frequent, only two died from cancer. All the pituitary tissues showed no histological alterations, nor molecular alterations.

### 3.2. Pituitary Adenomas Proteome Profile from POU1F1-Cell Lineages

**Figure 1:**
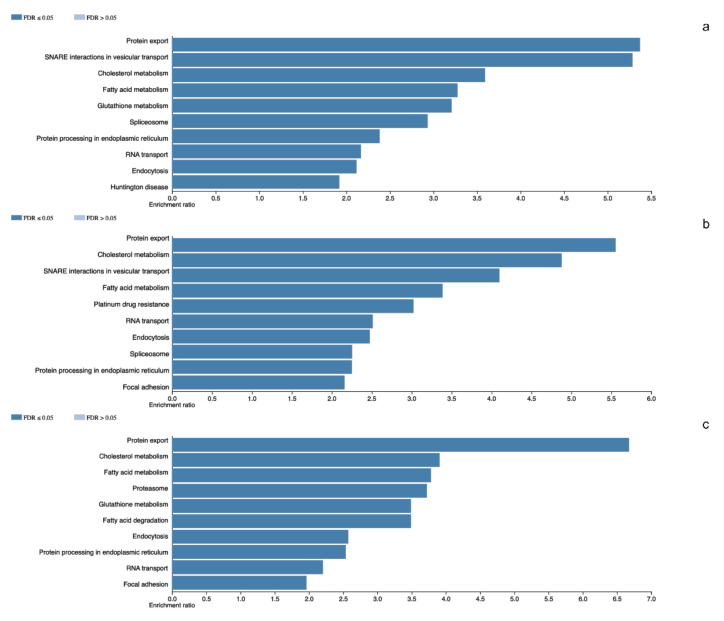
Panel (**a**–**c**) denotes the altered cellular events in which total proteins participates in *NR5A1*- and *POU1F1*-derived pituitary tumors and total detected proteins in control glands, respectively.

In the tumors corresponding to this group we identified a total of 2824 different proteins. The tissue with the least diversity contains 1343 different proteins whilst the most diverse contains 2012 different proteins. We carried out the identification of the altered cellular events. Spliceosome (*RBMX, SRSF3* and *U2AF1*) was observed as one of the representative events in tumor molecular machinery. Moreover, basic cellular events such as Protein export (*SPCS1, SRP14* and *SEC62*), Cholesterol metabolism (*APOA1, SOAT1* and *NCEH1*) and Focal adhesion (*CAV1, ILK* and *RHOA*) were observed (Figure 1).

### 3.3. Pituitary Adenomas Proteome Profile from NRD5A1-Cell Lineages

The analyzed NFPA showed a total of 3060 different proteins. Among these, 1279 different proteins were the least diverse whereas 2121 were the most diverse set of proteins. Once more, we observed in these tumors the Spliceosome (*SRSF1, U2AF2 and PRPF3*) as a component of the molecular machinery of these tumors. Among other altered cellular processes are Protein export (*SEC63, SRPRB* and *SRP72*), Fatty acid metabolism (*ACACA, CPT1A* and *MCAT*) and Glutathione metabolism (*GPX1, GSTK1* and *GSTO1*) among others (Figure 1).

### 3.4. Non-Tumoral Pituitary Proteome

A total of 2453 different proteins were observed in the non-tumoral gland, with 1612 in the tissues with the least protein diversity and 2002 in the tissue with highest diversity of proteins. Proteasome (*PSMD1, PSME6* and *ADRM1*) was one of the most representative events in these tissues. Among other processes RNA transport (*EIF1, NUP62* and *XPO1*), and Endocytosis (*AP2A1, CAV1* and *SNX12*) were observed (Figure 1).

### 3.5. Similarities and Differences between Pituitary Tumors and Non-Tumoral Gland Proteomic Profiles 

**Figure 2:**
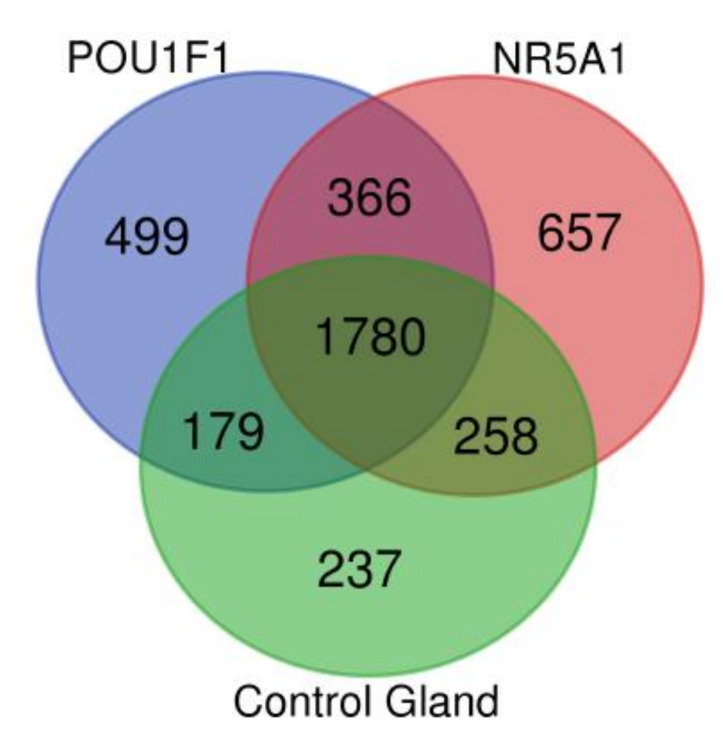
Venn diagram showing the common and distinctive proteins identified in the two pituitary tumor lineages and control gland.

**Figure 3:**
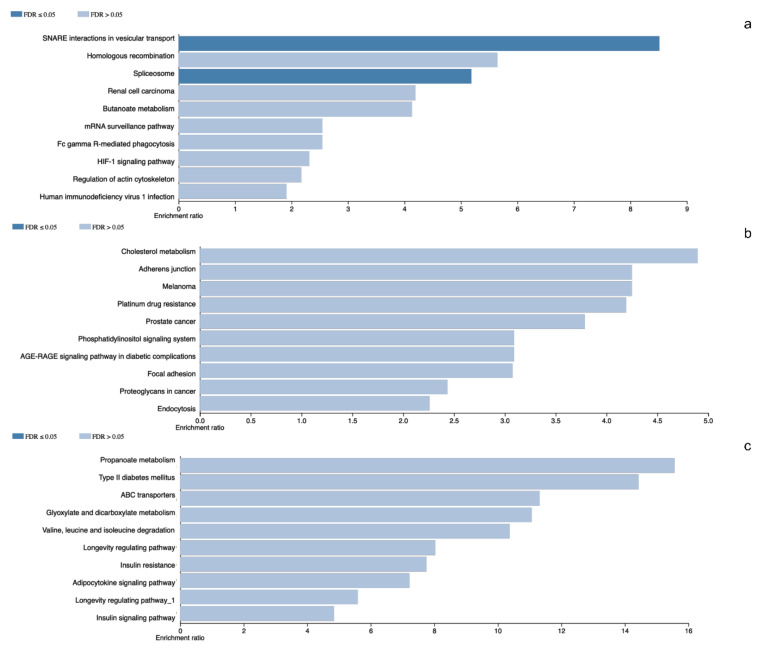
Panel (**a**–**c**) represents gene ontology results from the differentially identified and characteristic proteins in *NR5A1*- and *POU1F1*-derived pituitary tumors and control gland, respectively.

The pituitary adenomas derived from *POU1F1*- and *NR5A1*-cell lineages as well as the non-tumoral gland share a total of 1780 proteins (Figure 2). They also share several of the cellular processes such as Protein export, Cholesterol metabolism and Fatty acid metabolism among others. This possibly points to a common tissue origin of the tumors. The *POU1F1*-derived tumors have 499 exclusive proteins that participate in events such as Platinum drug resistance (*AKT3, BCL2* and *GSTT2*) and Prostate cancer (*FGFR1, IGF1R* and *PDGFD*) among others. The *NR5A1*-derived tumors showed 657 unique proteins that participate in Renal cell carcinoma (*CRK, ELOB* and *PAK3*) and Homologous recombination (*ATM, BRCC3* and *MRE11*) among others. Finally, the non-tumoral gland display 237 unique proteins participating in Insulin signaling pathway (*INSR, ISR2* and *mTOR*) and Propanoate metabolism (*HIBCH, PCCA* and *PCCB*) (Figure 3).

### 3.6. Whole Transcriptome mRNA Splicing Analysis

**Figure 4:**
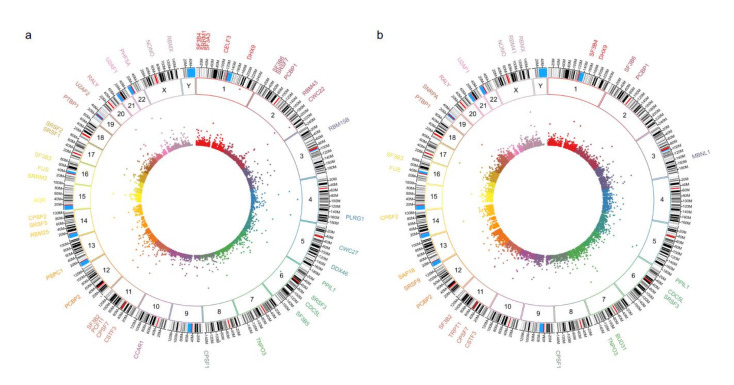
Panel (**a**,**b**) show the circos plot of the differentially mRNA expressed genes (inner circle), the chromosome (middle circle) and the proteins (outer circle) related to Spliceosome machinery or splicing events in *NR5A1*- and *POU1F1*-derived pituitary tumors, respectively.

**Figure 5:**
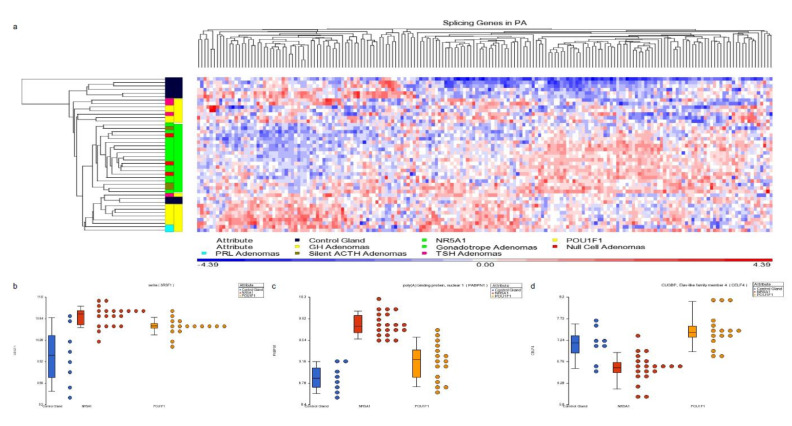
Panel (**a**) hierarchical cluster showing the spliceosome-related genes. NR5A1- and POU1F1-derived tumors show distinctive profiles which differentiate them using spliceosome-related genes. Panels (**b**–**d**) present dot blots from SRFS1 gene up-regulated in both cell lineages tumors, PABPN1 gene up-regulated predominantly in NR5A1-derived tumors and CELF4 gene up-regulated in POU1F1 tumors, respectively. Blue dots represent the non-tumoral control gland, red dots the NR5A1 tumors and yellow dots the POU1F1 tumors.

First, we validated our proteome findings at RNA level in a second cohort of *POU1F1*- and *NR5A1*- derived tumors. Several splicing-related genes were up-regulated at mRNA and protein levels (Figure 4). Genes such as *SRSF1* and *RBM42* were up regulated in both PA cell lineages, whereas genes like *PABPN1* were predominantly up-regulated in *NR5A1* derived tumors and *CELF4* predominantly was up-regulated in *POU1F1* derived tumors (Figure 5). Our results suggest that the PA could be segregated and categorized according to their cell lineages by the splicing-related genes profile (Figure 5).

**Figure 6:**
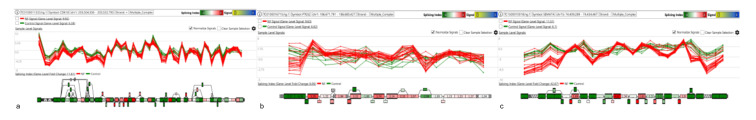
Panels (**a**–**c**) show the splicing events in *CDK18*, *PTGS2* and *SEMA7A* genes characterizing the *NR5A1* tumors.

**Figure 7:**
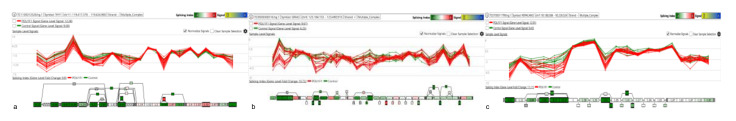
Panels (**a**–**c**) depict the splicing events in *THY1, GRIA3* and *HEPACAM2* genes characterizing *POU1F1*.

As a natural question raised by the pituitary tumor proteomic and transcriptomic profiles, we carried out the mRNA alternative splicing analysis. As expected, differentially expressed genes showed alternative splicing, that categorized and segregate the pituitary tumors into their specific cell linage. Splicing events in genes such as *CDK18, SEMA7A, PTGS2* and *KCNA3* characterize *NR5A1*-derived tumors, whereas splicing in genes such as *THY1, BMPER* and *GRIA3* characterize *POU1F1*-derived PA (Figure 6 and Figure 7).

Among the events identified in the alternative in gene splicing were predominantly cassette exon, alternative 3′ acceptor site, alternative 5′ donor site, and to a lesser extent intron retention, among others. 

**Figure 8:**
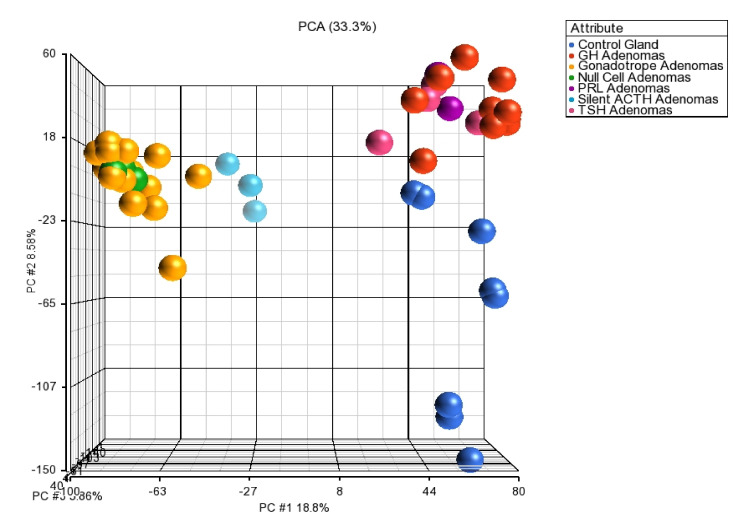
Principal component analysis of the pituitary tissue transcriptome. POU1F1-derived tumors GH-, TSH- and PRL-secreting adenomas clustering together as one group separated from the NR5A1-deived tumors gonadotropes, null cell and silent ACTH adenomas which comprised a separate cluster, both tumor lineages were isolated from control glands. Blue dots represent the control gland, red dots represent GH tumors, purple dots = PRL adenomas, pink dots = TSH adenomas, yellow dots = gonadotrope adenomas, green dots = null cell adenomas and cyan dots = silent ACTH adenomas.

The principal component analysis of the whole transcriptome, supports the results that molecular and cellular alterations are lineage-specific (Figure 8), thus correlating with the spliceosome and mRNA isoforms results that segregate and categorize each tumor cell lineage.

## 4. Discussion

Our results underscored the participation of spliceosome components in the molecular machinery of the pituitary tumors pathogenesis derived from *POU1F1*- and *NR5A1*-cell lineages and the alteration of the mRNA splicing patterns characteristic to each tumor. These tumor-specific spliceosome components and mRNA isoforms generated could support the notion that the pituitary tumors arise from divergent cell lineage origins or that they develop from partially committed pituitary stem cells, previously proposed by our group [10,11]. Similar results where the upregulation of several splicing factors in tumor-specific manner being observed in PA [12]. It is now established that alternative splicing contributes to cell differentiation and lineage determination, tissue identity acquisition and maintenance, and organ development [5]. The biological importance of alternative splicing is further highlighted by the large number of human diseases caused by alterations in *cis*-acting sequence elements in precursor mRNA (including 5ʹ and 3ʹ splice sites, and exonic and intronic enhancer or silencer sequences), *trans*-acting splicing factors or other components of the spliceosome [5]. Studies of tissue and organ development are particularly well suited to identifying the functional relevance of splicing networks, as the regulation of cell fate decisions is crucial for tissue and organ function and governs the transition from embryonic to adult functions [5]. The coordination of alternative splicing networks contributes to the development of various organs, maintaining tissue homeostasis and cell function [5]. Studies of the mechanisms of cell differentiation and dedifferentiation (transition from a differentiated state to a proliferative one) have also provided novel knowledge of the functional impact of splicing regulation [5]. Large-scale genomic studies have uncovered a spectrum of splicing machinery alteration that contribute to tumorigenesis [13]. Moreover, tumor cells are capable of hijacking the expression of RNA-binding proteins, leading to dysfunctional gene splicing and tumor-specific dependencies [13]. Advances in next-generation RNA sequencing have revealed tumor-specific isoforms associated with these alterations, including the presence of neoantigens, which serve as potential immunotherapeutic targets [13]. Functional alterations of this splicing-regulatory machinery can compromise the normal splicing process of an ample range of genes, thus originating the appearance of multiple, often aberrant, splicing variants, which could be directly associated with the development/progression of tumor pathologies [13]. Oncogenes have been shown to undergo isoform switching as a mechanism for cancer cells to escape certain therapies. Connections between dysregulated RNA splicing and tumor suppressor function have also been implicated in tumor biology [13]. Several of the genes that undergo alternative splicing found in our study are related to essential processes of the cell such as cell cycle, apoptosis and cell migration among others.

Pre-mRNA splicing is a common post-transcriptional process used by eukaryotic organisms to generate multiple transcript isoforms from a single gene. This process substantially expands the variety of encoded proteins, thus providing another means of functional regulation [14]. This process allows transcriptome and proteome diversification. Many transcripts are expressed in a tissue-specific manner and could also be disease- and developmental stage specific events. This suggests an evolutionary conserved molecular design for transcriptome diversification without the need to expand the genome [15]. Recent studies have shown that pre-mRNA splicing is involved in cancer [13]. Expression and activity of splicing factors are different among various types of cancer, which results in distinct splicing patterns of multiple transcripts that contribute to their pathophysiology involving processes such as cellular proliferation, migration and hormone responsiveness [16]. Splicing events in genes related to angiogenesis, cellular energetics, and avoiding the immune system response have shown participation in tumorigenesis [7].

The splicing factor *SRSF1* is involved in both, constitutive and alternative splicing, but also has additional functions such as regulating mRNA transcription, stability and nuclear export, as well as non-sense mediated mRNA decay and translation. It is frequently up-regulated in breast, colon, lung, thyroid, small intestine and ovarian tumors [17]. *SRSF1* participates in the pituitary tumor machinery regulating the isoforms generated from the *DIO1* gene [1]. *U2AF1* has been implicated in the altered splicing events in lung tumors, myelodysplastic syndrome and other hematological malignancies [1].

The splicing factor *RBM42* has been shown to be involved in the post-transcriptional regulation of genes involved in cell cycle progression [18] and ATP levels [19] and it is up-regulated in several tumors [20].

The *CELF* proteins participates in many aspects of gene expression, such as pre-mRNA splicing, RNA editing, deadenylation, mRNA stability and translations [21]. Its expression and functional activities in several tumors such as breast [22] and oral squamous cell tumors [23] have been reported.

From a mechanistic standpoint, the alteration of splicing factors must alter the downstream pattern of exon usage or splicing generated isoforms [24]. According to our results, PA not only harbor splicing-related proteins and mRNA alterations, but also, display abnormalities in the generated mRNA isoforms.

We found that genes that participate in important events for tumorigenesis could generate alternatively-spliced isoforms. Genes participating in cell cycle progression and genome stability such as *CDK18* [25] present potential isoforms in PA. Similarly, *THY1* genes which participate in T-cell activation, apoptosis, cell migration, cell adhesion and extravasation [26] also present splicing generated isoforms.

Spliceosome components and splicing-specific mRNA could be harnessed as molecular markers and therapeutic targets. Currently, there are several molecules targeting the spliceosome components that could be used in tumor therapy [27]. The identification of tumor specific mRNA’s could provide new opportunities for drug development. For instance, post-transcriptional dysregulation contributes to the antigen profile of tumors and this could be potentially leveraged for tumor therapy [28].

Discoveries in the past decade have highlighted the potential of mRNA processing as a therapeutic target for tumors. Although alternative splicing (AS) provides cells with a means to diversify the proteome, recent studies have revealed multiple ways by which splicing is pathologically altered to promote the initiation and/or maintenance of cancer [27]. These alterations result in numerous tumor-specific mRNAs that generate altered levels of normal proteins or proteins with new functions, leading to the activation of oncogenes or the inactivation of tumor suppressor genes [28]. Certain tumors are highly sensitive to the pharmacological inhibition of splicing [28]. Splicing is deregulated in tumors, leading to the expression of mRNA isoforms that have been implicated in tumor progression and invasiveness, cell proliferation, apoptosis, angiogenesis, metabolism and the cellular response to cancer treatment [28]. The identification of tumor-specific mRNAs that are generated by abnormal processing is providing new opportunities for the development of tumor therapies [28].

Over the last decades, multiple bacterially derived products and their analogs have been shown to bind spliceosome components and disrupt the early stages of spliceosome assembly [27]. These compounds which include Pladienolides, Herboxidienes, Spliceostatins, CLK inhibitors and SRPK inhibitors were identified as being potently cytotoxic and resulting in cell cycle arrest in the G1 and G2/M phases of the cell cycle [27]. Although a number of chemical compounds that modulate splicing catalysis have been described to date, nearly all of these drugs inhibit early spliceosome assembly or SR protein phosphorylation; however, it is quite likely that additional chemical screens will identify compounds that modulate the spliceosome at later stages of splicing catalysis [27]. Numerous preclinical models have suggested, somewhat unexpectedly, that modulating the activity of the spliceosome in general is well tolerated in vivo [27]. These studies have also suggested a potential rationale for the use of these compounds in tumor therapies [27].

## 5. Conclusions

In conclusion we present evidence supporting the participation of spliceosome components in PA molecular machinery. mRNA isoforms generated by alternative splicing appear to be specific for *POU1F1*- and *NR5A1*- derived pituitary tumors and could potentially be used as molecular markers as well as specific targets for molecular therapy.

## 6. Declarations

Ethics approval and consent to participate: All participating patients were recruited with signed informed consent and ethical approval from the Comisión Nacional de Ética e Investigación Científica del Instituto Mexicano del Seguro Social in accordance with the Helsinki declaration. 

Consent for publication Not applicable.

## Figures and Tables

**Table 1:** Clinical features of patients with *POU1F1*- and *NR5A1* derived pituitary adenomas.

	*POU1F1*	*NR5A1*	Non-Tumoral Gland
**Tumor size**			
Bigger than 10 mm	20	28	0
Smaller than 10 mm	2	0	12
**Gender**			
Male	6	17	4
Female	16	11	8
**Cavernous sinus invasion**			
Invasive	15	26	0
Non-invasive	7	2	12
**Recurrent**			
Recurrent	4	14	0
Non-recurrent	18	14	12

## Supplementary Materials

The following are available online at https://www.mdpi.com/2073-4425/11/12/1422/s1, Figure S1: Pituitary hormones and transcription factor immunohistochemistry. Availability of Data and Materials: The data presented in this study are openly available in Gene Expression Omnibus (GEO), which is hosted by the National Center for Biotechnology Information (NCBI) at accession number GSE147786.
