# Supplementary figures and images for: Proteomic and Transcriptomic Analysis Identify Spliceosome as a Significant Component of the Molecular Machinery in the Pituitary Tumors Derived from POU1F1- and NR5A1-Cell Lineages
Source: Genes (Basel). 2020 Nov 27;11(12):1422. doi: 10.3390/genes11121422 (PMC7760979; doi:10.3390/genes11121422)

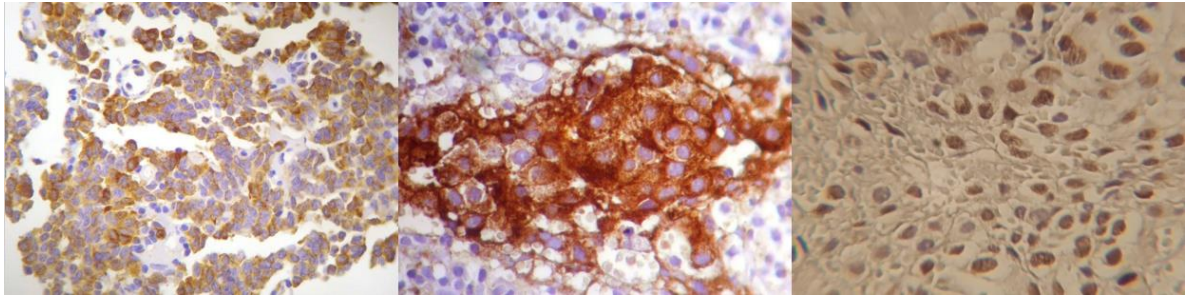

**Figure S1.** Pituitary hormones and transcription factor immunohistochemistry.

Supplement: Supplementary file 1 [file genes-11-01422-s001.pdf]
